# Un hémangiome intraosseux localement agressif de siège inhabituel

**DOI:** 10.11604/pamj.2019.32.114.17980

**Published:** 2019-03-11

**Authors:** Faten Limaïem

**Affiliations:** 1 Université de Tunis El Manar, Faculté de Médecine de Tunis, 1007, Tunisie

**Keywords:** Hémangiome, os, tumeur, vasculaire, Hemangioma, bone, tumor, vascular

## Abstract

Bone hemangioma is a benign primitive lesion characterized by the neoformation and the agglomeration of abnormal blood vessels. It most commonly involves the vertebrae and the craniofacial skeleton, but all of the bones may be injured. Astragalus involvement is rare. We here report the case of a 71-year old female patient with no particular past medical history, presenting with ankle pain evolving for two years. CT scan of the foot showed lytic lesion and associated fracture of the astragalus as well as a loss of substance inside the bone. Excisional biopsy was performed. Macroscopically this was a haemorrhagic friable material. Histologically, this was a benign vascular proliferation, consisting of vascular cavities, more or less filled with red blood cells and coated by a layer of flattened endothelial cells. There was no cytonuclear atypia or abnormal mitotic activity at the level of the endothelial cells. Immunohistochemically, these cells expressed the CD34. The diagnosis of bone hemangioma was made. Clinical and imaging manifestations of locally aggressive hemangiomas may be mistaken for a vascular or non-vascular malignant process. Histological examination is used to confirm the diagnosis. This tumor is generally stable, it sometimes regresses spontaneously in a few years. However, it may also have a locally aggressive evolution.

## Image en médecine

L'hémangiome osseux est une lésion primitive bénigne de l'os, caractérisée par la néoformation et l'agglomération de vaisseaux sanguins anormaux. L'atteinte des vertèbres et du squelette cranio-facial est la plus fréquente mais, tous les os peuvent être lésés. Sa localisation au niveau de l'astragale est rare. Il s'agit d'une patiente âgée de 71 ans sans antécédents pathologiques notables, qui avait consulté pour des douleurs de la cheville évoluant depuis deux ans. La tomodensitométrie du pied a objectivé une image lytique et une fracture de l'astragale ainsi qu'une perte de substance interne osseuse. Une biopsie chirurgicale de cette lésion a été pratiquée. Macroscopiquement, nous avons reçu un matériel hémorragique, friable. Histologiquement, il s'agissait d'une prolifération vasculaire bénigne, constituée de cavités vasculaires plus ou moins gorgées d'hématies et revêtues d'une assise de cellules endothéliales aplaties. Il n'y avait pas d'atypies cytonucléaires ni d'activité mitotique anormale au niveau des cellules endothéliales. Sur le plan immunohistochimique ces cellules exprimaient le CD34. Le diagnostic retenu était celui d'un hémangiome osseux. Les hémangiomes localement agressifs peuvent être confondus, cliniquement et à l'imagerie, avec un processus malin, vasculaire ou non. L'histologie apporte la confirmation diagnostique. Cette tumeur est généralement stable, parfois spontanément résolutive en quelques années. Elle peut cependant aussi avoir une évolution agressive localement.

**Figure 1:**
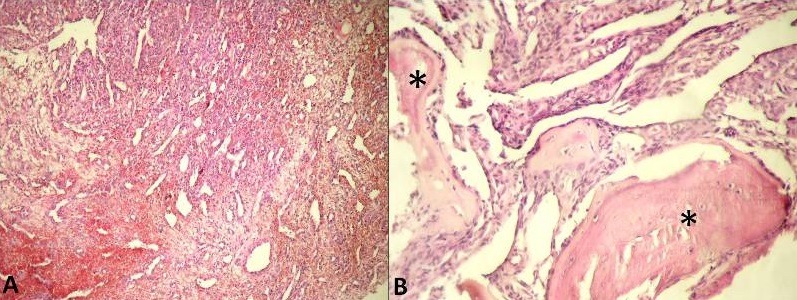
Le tissu osseux (astérix) est le siège d’une prolifération vasculaire bénigne, constituée de cavités vasculaires plus ou moins gorgées d’hématies et revêtues d’une assise de cellules endothéliales aplaties; il n’y avait pas d’atypies cytonucléaires ni d’activité mitotique anormale au niveau des cellules endothéliales (A) HE X 100, (B) HE X 400

